# Unlocking the Hidden Pathway: A Rare Encounter With Right Coronary Artery Fistula to Coronary Sinus and Right Ventricle

**DOI:** 10.7759/cureus.59155

**Published:** 2024-04-27

**Authors:** Marlon E Rivera Boadla, Nava R Sharma, Amit Gulati, Sakshi Khurana, Muhammad H Khan, Asiya Batool, Arafat Ali Farooqui, Juan S Cabrera, Triccia Aparicio Recarte, Arsalan Talib Hashmi

**Affiliations:** 1 Internal Medicine, Maimonides Medical Center, Brooklyn, USA; 2 Medicine, Manipal College of Medical Science, Pokhara, NPL; 3 Cardiology, Icahn School of Medicine at Mount Sinai, New York, USA; 4 Radiology, Columbia University, Brooklyn, USA; 5 Internal Medicine, Jinnah Hospital, Allama Iqbal Medical College (AIMC), Lahore, PAK; 6 Medicine, Universidad Tecnológica Centroamericana (UNITEC), Tegucigalpa, HND; 7 Medicine, Universidad Nacional Autonoma de Honduras, San Pedro Sula, HND; 8 Cardiology, Maimonides Medical Center, Brooklyn, USA

**Keywords:** coronary sinus (cs), exertional chest pain, cardiology cardio intervention cardiac imaging, coronary artery fistula (caf), coronary arterial fistula

## Abstract

Coronary artery fistulas (CAFs) are rare vascular anomalies characterized by abnormal connections between coronary arteries and cardiac chambers or adjacent structures. Advances in cardiac interventions have led to an increasing recognition of acquired CAFs, which are typically congenital. We present a case of a 62-year-old male with a complex medical history, including hypertension, atrial fibrillation, and heart failure, who presented with exertional chest pain and palpitations. Diagnostic evaluation revealed a significant CAF originating from the right coronary artery (RCA) and terminating into the coronary sinus and right ventricle. Despite the absence of significant coronary artery occlusions, the fistula was deemed clinically significant due to its potential to cause myocardial ischemia. Management involved guideline-directed medical therapy and lifestyle modifications. This case underscores the importance of early recognition and appropriate management of CAFs to optimize patient outcomes. Further research is needed to better understand the natural history and optimal management strategies of CAFs.

## Introduction

Coronary artery fistulas (CAFs) are uncommon anomalies seen in 0.1% to 0.2% of the general population [1]. They are characterized by abnormal connections between coronary arteries and cardiac chambers, adjacent veins, or mediastinal structures [2]. While typically congenital, acquired CAFs have become more prevalent with the rise in cardiac interventions and procedures. Although often asymptomatic, CAFs can lead to a spectrum of clinical manifestations, ranging from palpitations to myocardial ischemia and heart failure [3]. Diagnosis is frequently incidental and often discovered during routine cardiac imaging studies.

We present a case of a 62-year-old man with a complex medical history including hypertension, atrial fibrillation, and heart failure with reduced ejection fraction who presented with symptoms of exertional chest pain and shortness of breath. Subsequent evaluation revealed a significant coronary artery fistula originating from the right coronary artery (RCA) and terminating into the coronary sinus and right ventricle, highlighting the importance of recognizing such incidental anomalies in clinical practice.

## Case presentation

A 62-year-old male presented to the hospital with a known history of hypertension, atrial fibrillation, and heart failure with a reduced ejection fraction. The patient reported experiencing intermittent palpitations over the past several months, which had become increasingly bothersome. Additionally, he noticed numbness in his left middle finger that persisted for two months. However, he denied any chest pain or shortness of breath at the time of presentation.

**Figure 1:**
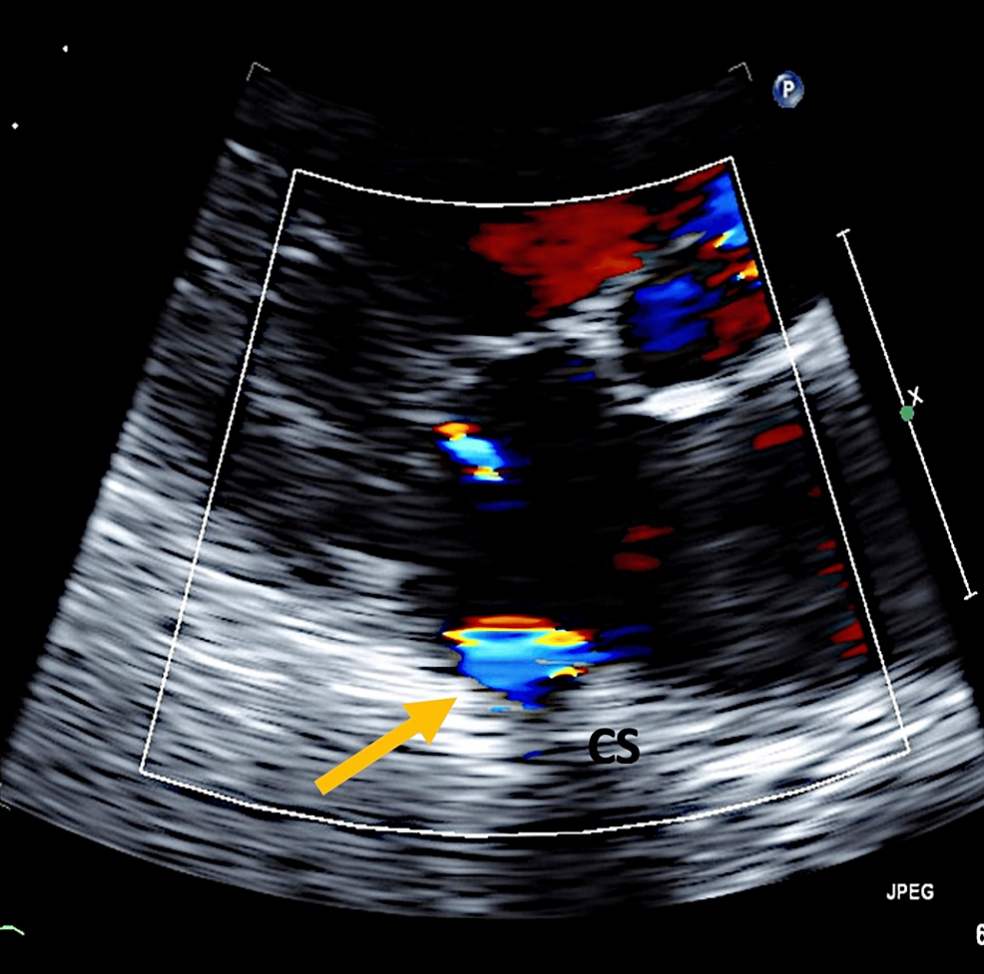
Echocardiogram showing coronary sinus fistula to the right ventricle (yellow arrow). CS: coronary sinus.

Upon admission, the patient's vital signs were stable, with a blood pressure reading of 141/65 mmHg. Physical examination revealed no significant abnormalities apart from signs of left ventricular hypertrophy (a palpable strong point of maximal impulse with possible lateral and downward displacement, voltage criteria on EKG, as well as thickened left ventricular walls observed on echocardiography). Laboratory investigations revealed elevated troponin levels, initially measuring 2.13 ng/mL and subsequently peaking at 5.9 ng/mL (normal reference: 0 to 0.04 ng/mL), suggestive of myocardial injury. The initial echocardiography showed signs of coronary sinus fistula, as shown in Figure 1. While color flow in the coronary sinus was noted on echocardiography, this finding alone is not diagnostic of CAF and can be observed in various scenarios, such as persistent left-sided superior vena cava.

**Figure 2:**
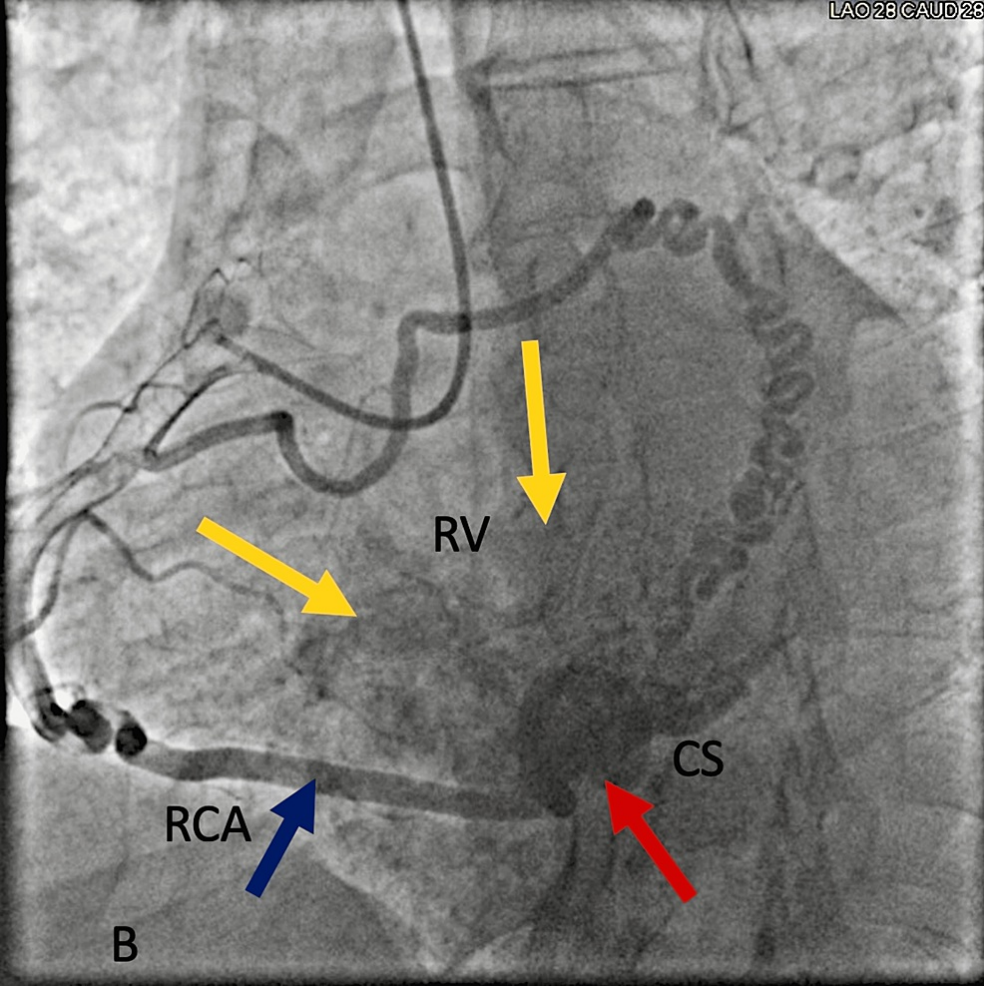
Coronary angiogram shows contrast flowing to the coronary sinus (CS, red arrow) and right ventricle (RV, yellow arrows) from the right coronary artery (RCA, blue arrow).

Further diagnostic workup included a stress test, which revealed a fixed inferior wall perfusion defect, raising concerns for underlying coronary artery disease. Subsequently, coronary angiography was performed to assess coronary artery patency. The coronary angiogram showed no occlusion in the coronary arteries but was remarkable for a large coronary artery to coronary sinus fistula extending to the right ventricle, as shown in Figure 2. Continuous turbulent flow was noted in the coronary sinus, and the coronary arteries appeared dilated (ecstatic).

Given the patient's clinical presentation and imaging findings, a diagnosis of coronary artery fistula was established. Despite the absence of significant coronary artery occlusions, the fistula was deemed clinically significant due to its potential to cause myocardial ischemia and other complications. Therefore, the patient underwent left heart catheterization, which confirmed the presence of the coronary artery fistula.

Following the diagnostic procedures, the patient was started on guideline-directed medical therapy to manage his cardiac symptoms and optimize cardiovascular function. The treatment plan also included lifestyle modifications, such as dietary changes, exercise regimens, and smoking cessation, to promote long-term cardiovascular health.

The patient's response to treatment was favorable, with improvements noted in his symptoms, including a reduction in palpitations and chest discomfort. He expressed satisfaction with the management plan and was encouraged to adhere to the prescribed treatment regimen. Outpatient follow-up appointments were scheduled to monitor his progress and assess the efficacy of the treatment interventions.

## Discussion

Coronary artery fistulas (CAFs) represent rare congenital or acquired vascular anomalies characterized by abnormal connections between coronary arteries and cardiac chambers, adjacent veins, or mediastinal structures. The right coronary artery (RCA) is the predominant site of origin for CAFs, constituting approximately 50% to 55% of all cases [4].

The pathogenesis of congenital CAFs is multifactorial and not entirely understood, with proposed mechanisms including failure of proper coronary artery development during embryogenesis or abnormal involution of primitive myocardial sinusoids [5]. Acquired CAFs, on the other hand, may arise secondary to cardiac interventions (e.g., cardiac surgery, percutaneous coronary interventions), myocardial biopsy, or trauma [6].

Clinical presentation of CAFs varies widely, ranging from asymptomatic cases incidentally detected during routine cardiac imaging to symptomatic cases presenting with palpitations, chest pain, dyspnea, or signs and symptoms of heart failure [3,6,7]. In severe cases, CAFs may lead to myocardial ischemia, arrhythmias, or ventricular dysfunction, necessitating prompt diagnosis and intervention [3,8]. One of the most common manifestations is chest pain due to myocardial ischemia due to a steal phenomenon in which the high-pressure coronary arterial flow drains into a lower-resistance venous circuit through the fistula without flowing through the myocardial capillaries, creating a decreased perfusion in the distal site of the fistula [9].

Diagnostic evaluation typically involves a combination of noninvasive imaging modalities such as echocardiography, computed tomography, magnetic resonance imaging, and invasive angiography. Coronary angiography remains the gold standard for confirming the presence of CAFs, delineating their anatomical characteristics, and assessing hemodynamic significance [10]. Angiographic findings may include tortuous vessel course, dilated feeding arteries, and drainage into cardiac chambers or adjacent structures [10].

Management of CAFs is individualized and depends on several factors, including the size, location, and hemodynamic consequences of the fistula, as well as the presence of symptoms. While small, asymptomatic fistulas may be managed conservatively with regular surveillance, larger or symptomatic fistulas may warrant closure to prevent complications such as myocardial ischemia, heart failure, or infective endocarditis [10,11].

Surgical ligation or transcatheter closure using occlusion devices is a commonly employed technique for closing significant CAFs. However, closure of fistulas originating from the right coronary artery presents a unique challenge due to the risk of proximal RCA dilatation and subsequent myocardial infarction post-closure. Therefore, careful consideration of the risks and benefits is essential in such cases [1,10,12].

In our presented case, a 62-year-old male with a history of hypertension, atrial fibrillation, and heart failure with reduced ejection fraction, presented with exertional chest pain and shortness of breath. Diagnostic evaluation revealed a significant coronary artery fistula originating from the RCA and terminating into the coronary sinus and right ventricle. Following confirmation of the diagnosis through left heart catheterization, the patient was managed with guideline-directed medical therapy and lifestyle modifications.

## Conclusions

In summary, this case highlights the importance of recognizing and managing CAFs. Our patient, a 62-year-old male with a complex medical history, presented with exertional chest pain and shortness of breath, ultimately diagnosed with a significant CAF originating from the RCA. Diagnostic evaluation confirmed the presence of the fistula, prompting guideline-directed medical therapy and lifestyle modifications. While further research is needed to better understand CAFs, this case underscores the significance of early detection and appropriate management in optimizing patient outcomes.
